# An economic evaluation of the Whole Genome Sequencing source tracking program in the U.S.

**DOI:** 10.1371/journal.pone.0258262

**Published:** 2021-10-06

**Authors:** Brad Brown, Marc Allard, Michael C. Bazaco, Joseph Blankenship, Travis Minor

**Affiliations:** United States Food and Drug Administration, Center for Food Safety and Applied Nutrition, College Park, Maryland, United States of America; Cornell University, UNITED STATES

## Abstract

The U.S. Food and Drug Administration (FDA) created the GenomeTrakr Whole Genome Sequencing (WGS) Network in 2013, as a tool to improve food safety. This study presents an analysis of Whole Genome source tracking implementation on potential food contamination and related illnesses through theoretical, empirical, and cost benefit analyses. We conduct empirical tests using data from FDA regulated food commodity outbreaks garnering FDA response from 1999 through 2019 and examine the effect of the National Center for Biotechnology Information (NCBI) Pathogen detection program of source tracking WGS isolates collected in the U.S. on outbreak illnesses for three pilot pathogens (*E*. *coli*, *Listeria*, and *Salmonella*). Empirical results are consistent with the theoretical model and suggest that each additional 1,000 WGS isolates added to the public NCBI database is associated with a reduction of approximately 6 illnesses per WGS pathogen, per year. Empirical results are connected to existing literature for a Monte Carlo analysis to estimate benefits and costs. By 2019, annual health benefits are estimated at nearly $500 million, compared to an approximately $22 million investment by public health agencies. Even under conservative assumptions, the program likely broke even in its second year of implementation and could produce increasing public health benefits as the GenomeTrakr network matures.

## Introduction

Despite significant effort to improve and modernize the food safety system in the United States, foodborne pathogens remain a major public health threat, causing an estimated 9.4 million illnesses each year, including 56,000 hospitalizations, and 1,400 deaths [1]. In the United States, the regulation of food safety is primarily divided among two government agencies, the U.S. Food and Drug Administration (FDA) and the U.S. Department of Agriculture Food Safety Inspection Service (USDA-FSIS). Illnesses caused by foods regulated by the FDA account for roughly 80% of the total estimated annual foodborne illnesses in the U.S. [2]. The FDA’s Center for Food Safety and Applied Nutrition (CFSAN) created the GenomeTrakr Whole Genome Sequencing (WGS) Network in 2013, as a tool to help improve food safety [3, 4]. To date, the GenomeTrakr network is made up of over 50 national and international laboratories that are sequencing foodborne pathogens and uploading the genomes into the National Center for Biotechnology Information Pathogen Detection (NCBI PD) web portal. Other national and international public health authorities also share their WGS data at NCBI. Daily phylogenetic clusters are generated at NCBI documenting new and emerging linkages of possible contamination. Compared to Pulsed-field Gel Electrophoresis (PFGE), WGS provides more precise, high resolution source tracking and predictions for food and environmental genomic data [5]. Results from the analysis of the data enables faster and more precise public health and regulatory actions (such as public messaging and recalls), thus decreasing the number of illnesses associated with outbreaks and decreasing the breadth of products recalled on average [6, 7]. The program makes it easier to evaluate cases across time and geography, helping to solve ongoing contamination events that would otherwise go unidentified [8–10] The use of WGS data increases the effectiveness in monitoring and FDA’s ability to perform root cause analysis, providing information to growers and manufacturers of food that can help them make effective investments and improvements in their food safety systems [11–13].

FDA along with CDC, USDA-FSIS, NCBI, and state and territorial public health agencies adopted WGS from 2013–2019 to replace PFGE as the preferred subtyping method for use in PulseNet [14]. The benefits of PulseNet’s adoption of PFGE were previously estimated at approximately half a billion dollars annually [5]. Higher resolution technology like WGS should provide even greater economic benefit.

Previous studies have analyzed the effects of similar programs. Scharf et al. (2016) estimated the economic benefits of PulseNet, the predecessor and coexisting program to NCBI PD, using information from two representative outbreaks and recalls [15]. In total, they estimated that PulseNet resulted in benefits of over $540 million annually in averted illnesses. Jain, et al. (2019) estimated the effect of the Canadian WGS program [16]. They estimated net benefits of the Canadian WGS surveillance and source tracking program on *Salmonella* alone to be between $5 -$90 million. This study, relying on a more complete data set on *Salmonella* and two additional pathogens, *Listeria* and *E*. *coli* (primarily virulent STEC, due to its importance in public health) should provide a more accurate picture of the impact of the use of WGS data and the NCBI PD for more effective source tracking and surveillance in the U.S.

In this paper, through theoretical, empirical, and cost benefit analyses, we evaluate the effects of the NCBI PD program (of which GenomeTrakr is a part of that collaboration) on the FDA’s ability to detect, investigate, and limit the spread of outbreaks linked to FDA-regulated commodities, and the costs of the program. Specifically, we provide a fully specified benefit-cost analysis of the program based on three of the pilot pathogens’ (*E*. *coli*, *Listeria*, and *Salmonella*) unique isolates and their estimated impact on public health outcomes [3, 6]. This information is then coupled with costs of implementation of the WGS network to better understand the net-benefits or costs of this public safety network and data collection.

## Materials and methods

To examine the currently realized benefits and costs of the WGS NCBI PD program in the US we employ a multi-tiered analysis. First, using an established theoretical economic framework, we model the expected implications of the WGS NCBI PD program on consumer health as well as industry and government expenditures. Next, we empirically test the theoretically ambiguous effect of the WGS NCBI PD program on human foodborne illnesses using regression analysis on a novel database assembled for this purpose. Finally, utilizing results from the regression analysis, as well as other published literature, we fully parameterize the theoretical model to generate annual benefits and costs of the WGS NCBI PD program to date. The remainder of this section goes through each of these distinct steps in detail.

### Theoretical model

Eq 1 presents a theoretical model illustrating the potential effects of the WGS program and sets the stage for the empirical and benefit/cost analyses, using a social welfare maximization framework of goods production with an externality first developed by A.C. Pigou and commonly used in modern welfare and environmental economics [17, 18]. The social value function (*SV*) and the potential effects of the WGS program in the US are modeled as the net value of food production (profit function), minus the total burden of foodborne illness associated with food production (public health externality function), minus the implementation costs of the program. The full derivation of the model and isolated effects are shown in the S1 Appendix.


$$SV=\underset{profit\;Function}{\underbrace{\left[p_{x}*x-\left(c_{x}\left(x\right)+c_{e}\left(e(WGS\right)\right)\right]}}-\underset{public\;health\;externatility\;function}{\underbrace{\left[C_{I}*x*\gamma_{I}\left(e\left(WGS\right)\right)*n_{I}\left(WGS\right)\right]}}-\underset{implentation\;cost}{\underbrace{\left[c_{WGS}\left(WGS\right)\right]}}$$ (1)

The profit function of the representative firm captures the value of the goods produced to industry and consumers. A representative firm maximizes profits over production of *x* with the constant per unit consumer price *p*_*x*_ and the costs of production *c*_*x*_(*x*) that are a function of how much they produce and the unit costs of production. The firm also invests in food safety controls, *e*. As the firm invests more, the effectiveness of the controls increases. The cost of investment in controls *c*_*e*_(*e*(*WGS*)) is increasing in *e*, and likewise, *e*(*WGS*), is an increasing function of *WGS*.

The public health externality function captures effects not fully internalized by the profit maximizing firm, in this case, the potential effects of foodborne illness associated with food production. In the model, *γ*_*I*_(*e*(*WGS*)) is the probability for any level of *e(WGS)*, that a unit of production causes an outbreak. The probability decreases as the firm increases its investment in food safety controls. The number of illnesses associated with an outbreak, *n*_*I*_(*WGS*), is a decreasing function of *WGS*, and *C*_*I*_ is the marginal burden of illness.

Finally, the variable, *c*_*WGS*_(*WGS*), is the direct implementation cost of WGS source tracking.

Taking the partial derivative of the SV function with respect to WGS shows that WGS source tracking has four primary effects on the social value function. On the cost side, WGS source tracking informs firms of potential contamination vectors [11–13], and provides an incentive to increase investment in control effectiveness [7]. WGS source tracking also has direct implementation costs. On the benefits side, shown as part of the externality function, WGS source tracking will decrease the cost of outbreaks or illness occurrences by facilitating faster, more efficient tracing of the sources of contamination, decreasing the number of illnesses in outbreaks [6]. Further, the change in investment in effective food safety controls, due to WGS source tracking implementation, will affect the probability of an outbreak or illness event.

In total, the net effect of WGS source tracking on social welfare is ambiguous. We cannot know without further analysis, weighing the benefits of illness reduction against the costs of implementation, whether the WGS program in the US provides a net-benefit or cost as a whole (i.e., to consumers, industry, and government). In the early stages of implementation of any program, it is possible the costs could outweigh the benefits.

Early adoption of WGS source tracking could drive results of an empirical analysis in opposite directions. WGS source tracking will help identify more illnesses associated with outbreaks, so the probability of detection should increase [3]. Additionally, WGS source tracking will identify outbreaks in smaller clusters, decreasing the average size (average number of illnesses) of observed outbreaks [6].

Drawing from Eq 1, the number of observed illnesses, *I*_*O*,_ is the product of the total number of illnesses and the probability that an illness is observed, defined as *α*_*O*_(*WGS*). The observed illness/externality function becomes:

IO=x*γIeWGS⏟probabilityoutbreakoccurs*nIWGS⏟numberofillnessesinoutbreak*αO(WGS)⏟probabilityillnessesareobserved (2)

Taking the partial derivative to isolate the effect of WGS source tracking shows the net effect on the number of observed illnesses is indeterminant. The effect of WGS source tracking on outbreak probability and the number of illnesses per outbreak should be negative [4]. However, the effect on the probability that an illness is detected is positive [3]. If the effect of WGS on the probability of all illnesses occurring dominates, illnesses will decline. However, if the effect of WGS on detection dominates, observed illnesses will increase even as the total number of illnesses falls.

### Empirical model

The data for this analysis is primarily extracted from the FDA’s Coordinated Outbreak Response and Evaluation (CORE) database and data on pre-CORE outbreaks investigated by FDA from 1999–2019, prior to the initiation of CORE. The CORE database includes detailed information on foodborne outbreaks that were investigated by the FDA. While this data does not represent all outbreaks related to (or likely associated with) FDA-regulated human foods, it does represent the scope of outbreaks with direct FDA involvement. The database includes information on the number of confirmed illnesses, the associated pathogen and food vehicle, and the timing of FDA’s investigations for each outbreak. From this data, we construct a balanced pathogen and year panel comprised of the annual number of illnesses and outbreaks for each observed pathogen between 1999–2019. The extended time-period and inclusion of non-sequenced pathogens allows for more robust estimates, accounting for trends and variation that may be present in the data prior to National Center for Biotechnology Information Pathogen Detection program implementation [3, 6, 19]. The final, balanced panel-database is available in the S1 Appendix to this analysis.

Additional data on WGS source tracking isolates is drawn from the NCBI PD database. NCBI collects sequencing data submitted by public health officials, academic researchers, or industry sources as a central repository designed to facilitate analysis and aid in outbreak and traceback investigations. This tool is used by epidemiologists and other bioinformaticians to recognize clusters of interest and link any clinical isolates that may be genetically related to other clinical, food, or environmental isolates within the existing database more quickly. As the database grows, this predictive power is improved, thus facilitating interventions that may help to curb foodborne illness. We use the annual number of unique WGS isolates compiled by NCBI as a proxy for the maturity of the program’s library of genomes and thus the potential preventive power of this program [3, 6, 20]. Because we are less interested in the sequences themselves than the predictive power of the entire library as it expands, we utilize NCBI’s own inclusion criteria as a minimal threshold for inclusion in our database [20].

Because the data on WGS source tracking is relatively limited, with widespread collection only beginning around 2014, we construct a panel of pathogen/year data to examine WGS source tracking and foodborne illnesses and to tease out any effects within the data. Initial estimation in Model 1 takes the form:

$$Y_{p,t}=\beta_{0}+{\beta_{1}WGS\_library}_{p,t}+\varepsilon_{p,t}$$ (3)

Where *Y*_*p*,*t*_ is a measurement of foodborne illness specified as either (1) observed outbreak illnesses for pathogen *p* at time *t*, (2) observed outbreaks for pathogen *p* at time *t*, or (3) the average number of illnesses per outbreak for pathogen *p* at time *t*; *β*_0_ is the intercept; *WGS_library*_*p*,*t*_ is a measure of the number of WGS isolates uploaded to NCBI’s repository for pathogen *p* at time *t* (in 1,000s); and *ε*_*p*,*t*_ is the idiosyncratic error term. *β*_1_ is the coeffect of interest, as it captures the change in observed *foodborne illness* outcomes directly correlated with the number of WGS isolates available per pathogen.

In addition to the effects of WGS source tracking, there are a limited number of variables that may affect illness and outbreak occurrence in a given year that are recorded in the CORE database. We collect information on the pathogen implicated (*Pathogen Indicators*), the food vehicle identified (*Food Vehicle*), the year in which the outbreak was investigated. Each of the food vehicles are initially recorded as a 0/1 indicator variable at the outbreak level, and then combined within time periods. Model 2 adds Food Vehicle indicators as well as Pathogen Indicators for all observed pathogens. Estimation takes the form:

$$Y_{p,t}=\beta_{0}+{\beta_{1}WGS\_library}_{p,t}+{\beta_{2}X}_{p,t}+\varepsilon_{p,t}$$ (4)

Where all variables are specified as before except *X*_*p*,*t*_ represents a series of time- and pathogen-variant controls (including pathogen fixed effects, year fixed effects, and food vehicle status). *β*_1_ remains the coeffect of interest.

Finally, there are confounding factors that may impact the measurement of WGS source tracking on foodborne illness. Specifically, in this analysis we are concerned with the confounding effects of the proposal and implementation of the Food Safety Modernization Act (FSMA) on our measured outcome. FSMA was signed into law in 2011, however the first proposed rules, with actionable items for food producers spanning the food supply to implement, were not published until 2013. The final Preventive Controls Rule and Produce Safety Rule were published in 2015, with implementation dates for the largest operators one year later [21]. Thus, we create indicator variables to capture each of these policy changes. Model 3 adds controls for the implementation of FSMA. Estimation takes the form:

$$Y_{p,t}=\beta_{0}+{\beta_{1}WGS\_library}_{p,t}+{\beta_{2}X}_{p,t}+{\beta_{3}FSMA}_{t}+\varepsilon_{p,t}$$ (5)

Where all variables are specified as before except *FSMA*_*t*_ which is comprised of three indicator variables that control for the proposal, finalization, and implementation of FSMA rules across all pathogens. *β*_1_ remains the coeffect of interest.

Regression analysis is performed using the ‘regress’ command in Stata (version 16.1) software, with robust standard errors clustered at the pathogen level; relevant code is provided in the S1 Appendix. The data and estimation methodology are similar to previous studies of public health interventions on foodborne illnesses [22–24]

### Benefit/cost model

To estimate the benefits of WGS source tracking we construct an analysis based on the estimated reduction outlined in the previous section. Benefits are constructed as:

$$Benefits={\hat{\beta }}_{1}\;x\;WGS\;Isolates\;x\;Underreporting\;Multiplier\;x\;Monetary\;Loss$$ (6)

For each pathogen, benefits are the product of the estimated marginal reduction in illness per 1,000 WGS isolates up, ${\hat{\beta }}_{1}$, thousands of WGS isolates in the NCBI PD library, an underreporting/underdiagnosis multiplier to capture the fact that not all illnesses are reported or diagnosed, and the estimated burden of illness related to each pathogen. Table 3 presents the parameters used in the estimation of benefits for this analysis. Dollar estimates from the literature are converted to 2019 constant dollars using the GDP deflator. Uncertainty distributions associated with the estimates are preserved or recreated.

Cost estimates include funds supplied by federal and state health agency partners, which capture lab set up costs, collection and testing costs, and internal costs to run the program. Monte Carlo Analysis is performed using @Risk (version 8) software. The simulation is run over 100,000 iterations. mean results as well as 90% confidence intervals are presented; a detailed methodology is provided in the S1 Appendix.

## Results

The results of this multi-tiered analysis of the benefits and costs of the WGS NCBI PD program in the US are laid out in detail below. First, we present the key summary statistics for the variables used in the empirical analysis. Next, we present results from the econometric analysis, formally estimating the effect of the WGS NCBI PD program on human foodborne illnesses using regression analysis. Finally, the results of the benefit/cost analysis, employing results from the econometric analysis as well as data from other relevant studies, are shown in full.

### Key summary statistics

Table 1 provides summary statistics for the variables used in the econometric analysis. Key outcome variables include the annual number of illnesses, outbreaks, and the average number of illnesses per outbreak. Annual illnesses average about 74.2 illness per year between 1999 and 2019, but there is a broad range from zero to 2,863 in a single year over all pathogens. The observed averages for this data are relatively low, due primarily to the fact that the data used for this analysis is not representative of overall foodborne illnesses but instead an indicator of outbreaks investigated by FDA during the time frame. Similarly, outbreaks average about 1.2 per year, ranging between zero and 38 in a single year, and average illnesses per outbreak average about 19.0 with a range between zero and 919.

**Table 1:** Summary statistics.

	Description	Mean	Standard Deviation	Variance	Min.	Max.	5^th^ Percentile	95^th^ Percentile
**Key Variables**								
Annual Illnesses	Illnesses at time t	76.94	286.46	82,058.12	0	2,863	0	494
Annual Outbreaks	Outbreaks at time t	1.29	3.57	12.73	0	38	0	6
Average Illnesses per Outbreak	Illness per Outbreak at time t	19.19	70.90	5026.13	0	919	0	104
**Whole Genome Sequence**								
WGS NCBI Library (1,000s)	Number of unique WGS isolates in the NCBI database as of Jan. 1, in thousands	0.74	6.54	42.83	0	111	0	0
**Pathogen Indicators (0/1)**								
*Listeria*	Equal to 1 for *Listeria* Observations	0.04	0.20	0.04	0	1	0	0
*E*. *coli*.	Equal to 1 for *E*. *coli*. Observations	0.04	0.20	0.04	0	1	0	0
*Salmonella*	Equal to 1 for *Salmonella* Observations	0.04	0.20	0.04	0	1	0	0
**Food Vehicles (0/1)**								
Dairy	Total number of outbreaks linked to dairy products at time t	0.08	0.41	0.17	0	4	0	1
Dietary Supplement	Total number of outbreaks linked to dietary supplements at time t	0.01	0.10	0.01	0	1	0	0
Egg	Total number of outbreaks linked to egg products at time t	0.24	2.15	4.64	0	29	0	0
Fish	Total number of outbreaks linked to fish products at time t	0.19	0.83	0.69	0	8	0	1
Infant Formula	Total number of outbreaks linked to infant formula at time t	0.01	0.11	0.01	0	2	0	0
Multiple Ingredients	Total number of outbreaks linked to multiple products at time t	0.04	0.22	0.05	0	2	0	0
Produce	Total number of outbreaks linked to produce products at time t	0.49	1.53	2.35	0	12	0	3
Shellfish	Total number of outbreaks linked to shellfish products at time t	0.11	0.49	0.24	0	4	0	1
Unknown	Total number of outbreaks not linked to a specific product at time t	0.05	0.27	0.07	0	3	0	0
**Food Safety Rules (0/1)**								
Proposed FSMA Rule	Equal to 1 in 2013 and beyond	0.33	0.47	0.22	0	1	0	1
Final FSMA Rule	Equal to 1 in 2016 and beyond	0.19	0.39	0.15	0	1	0	1
Implemented FSMA Rule	Equal to 1 in 2017 and beyond	0.14	0.35	0.12	0	1	0	1

NCBI, National Center for Biotechnology Information; FSMA, Food Safety Modernization Act; (0/1), indicator variable equal to 1 if true, zero otherwise.

Number of observations = 462. Year fixed effects are also included in the database. Each year has a mean of 0.05, a standard deviation of 0.21, and a variance of 0.5. Similarly, all pathogen indicators, including *Listeria*, *E*. *coli*., and *Salmonella*, will have the same summary statistics because the database is a balanced panel of pathogens (one observation per pathogen for each year of the data) over time.

**Fig 1:**
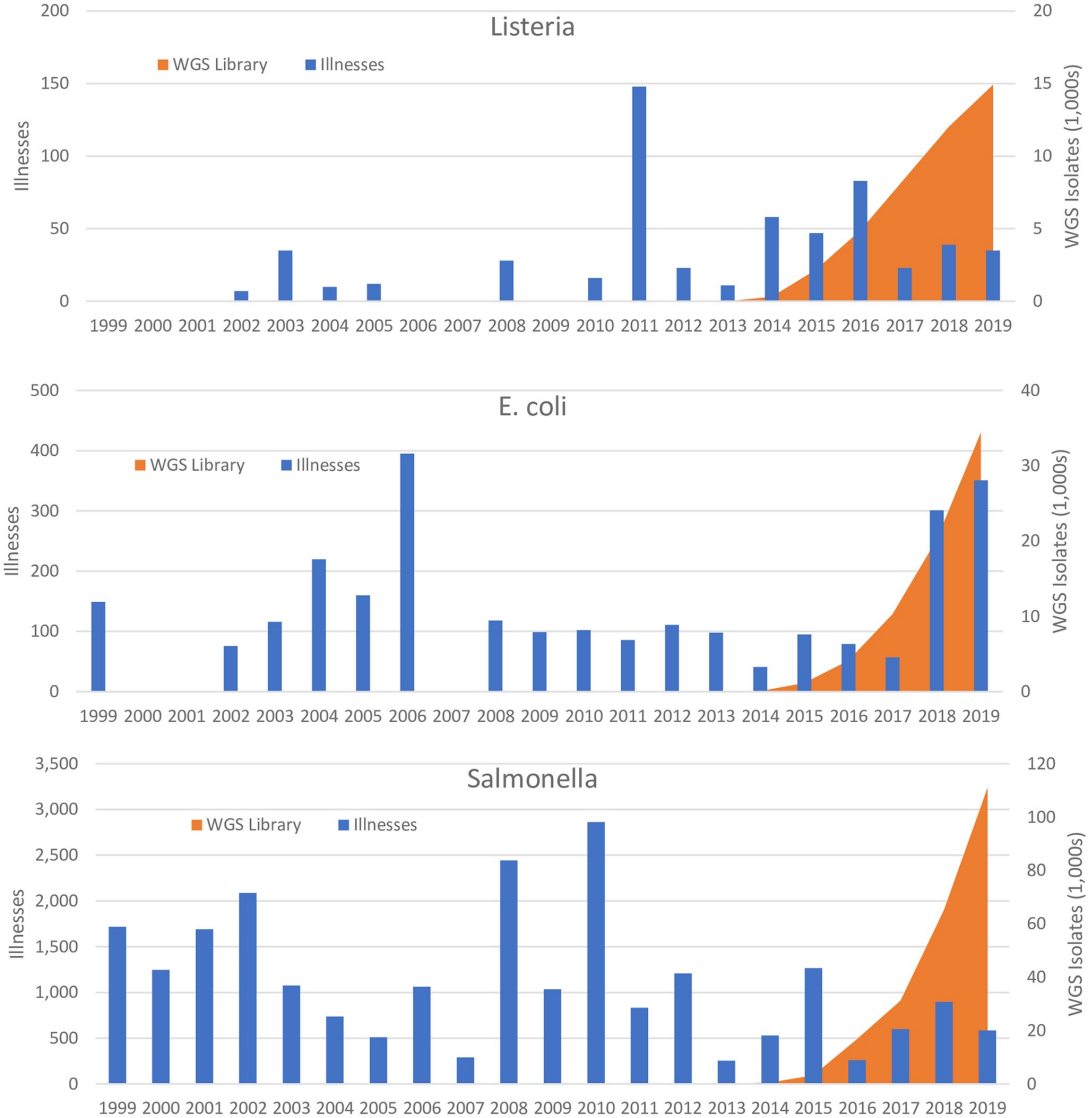
Illnesses & WGS isolates by pathogen. WGS, Whole Genome Sequence. Annual Illnesses for *Listeria*, *E*. *coli*., and *Salmonella* foodborne illnesses related to U.S. Food and Drug Administration regulated products charted against the number of unique isolates found in the National Center for Biotechnology Information’s database of sequenced isolates over time.

We measure the effects of the WGS program as the count of unique isolates in the NCBI PD library as of January 1 in a given year. The average number of unique WGS isolates for our three WGS pathogens over the observed time period is 740; however, this is skewed lower since collection did not begin in earnest until 2013. Fig 1 illustrates the relationship between annual illness and unique WGS isolates for the three pilot pathogens, *Listeria*, *E*. *coli*, and *Salmonella*, and shows that, while outbreak illnesses occur at differing magnitudes for all pathogens, WGS isolates follow a very similar trajectory after the establishment of the program. For all pathogens outside of *Listeria*, *E*. *coli*, and *Salmonella* WGS library will equal zero for all observations, because no WGS isolates exist in our examined time-period for any pathogens outside of these three initially piloted pathogens [3, 6].

Finally, the examined data in Table 1 shows observed outbreaks are more associated with produce (~47%) than any other commodity. This is somewhat distantly followed by egg products (~24%), fish (~19%), shellfish (~11%), and dairy products (~8%).

### The impact of the WGS library on foodborne illnesses

Table 2 presents the effect of increasing the WGS library (i.e. the number of domestic isolates publicly available at NCBI PD) on observed illnesses and outbreaks. Model 1, which estimates only the library on observed illnesses, suggests that each additional 1,000 WGS isolates of *Listeria* are associated with a statistically significant *increase* of 7.36 observed illnesses. Model 2, which adds the controls summarized in Table 1, estimates a statistically significant *reduction* of 6.11 fewer observed illnesses for each additional 1,000 isolates. Model 3, which adds controls for FSMA implementation, estimates a slightly smaller, statistically significant *reduction* of 6.09 observed illnesses for each additional 1,000 isolates added to the public library.

**Table 2:** Estimated effect of WGS library on illnesses and outbreaks.

	Annual Illnesses			Annual Outbreaks			Average Illnesses per Outbreak		
	Model 1	Model 2	Model 3	Model 1	Model 2	Model 3	Model 1	Model 2	Model 3
**WGS NCBI Library**	7.36***	-6.11**	-6.09**	0.14***	0.01*	0.01*	0.58**	-1.06***	-1.07***
	(0.67)	(2.25)	(2.25)	(0.01)	(0.01)	(0.01)	(0.21)	(0.32)	(0.31)
**R** ^**2**^	0.03	0.71	0.71	0.07	0.99	0.99	0.00	0.26	0.26
**Fixed Effect Controls**		X	X		X	X		X	X
**FSMA**			X			X			X

WGS, Whole Genome Sequence; NCBI, National Center for Biotechnology Information; FSMA, Food Safety Modernization Act.

Number of observations = 462.

Significance levels are indicated as:

*** significant at beyond the 1 percent level;

** significant at the 5 percent level;

* significant at the 10 percent level.

Fixed Effect Controls include Pathogen, Food Vehicle, and Year identifiers.

Examining outbreaks as the outcome of interest tells a different story. While Model 1 reports a statistically significant *increase* in observed outbreaks of about 0.14 for each 1,000 isolates, Models 2 and 3 suggest this effect, while still positive, is much smaller at a statistically significant 0.01 *more* outbreaks identified per 1,000 WGS isolates.

Finally, Table 2 presents some evidence that the WGS library is changing the makeup of outbreaks, by examining average illnesses per outbreak as an outcome. Model 1 suggests that increasing the WGS library by 1,000 isolates is associated with 0.58 *more* illness per outbreak. However, Models 2 and 3 suggest that increasing WGS isolates by 1,000 leads to approximately 1.07 *fewer* illnesses per outbreak.

Taken wholly, these results suggest that the WGS source tracking is associated with fewer illnesses for the collected pathogens over time, and this effect is largely observed because of smaller but slightly more frequent detection of outbreaks for those pathogens. Moving forward with the benefit-cost analysis, we focus on the estimated reduction in illnesses from Model 3.

### The benefits and costs of the WGS program

Table 4 presents estimated means and 90% confidence intervals for averted illnesses and associated monetary benefits, by year and pathogen. Estimates for reduction in illnesses due to WGS source tracking by 2019 range from 210 illnesses annually, or a 13% reduction for *Listeria*, the most heavily sequenced pathogen at this point, relative to the number of associated illnesses occurring each year, to roughly 19,800 illnesses, or about a 1.5% reduction for *Salmonella*, that has been sequenced relatively less and is more recently ramping up. The total burden of illness reduction in 2019 is nearly $500 million, or a little over 1.5% of total burden of illness attributed to FDA-regulated foods [25–27].

**Fig 2:**
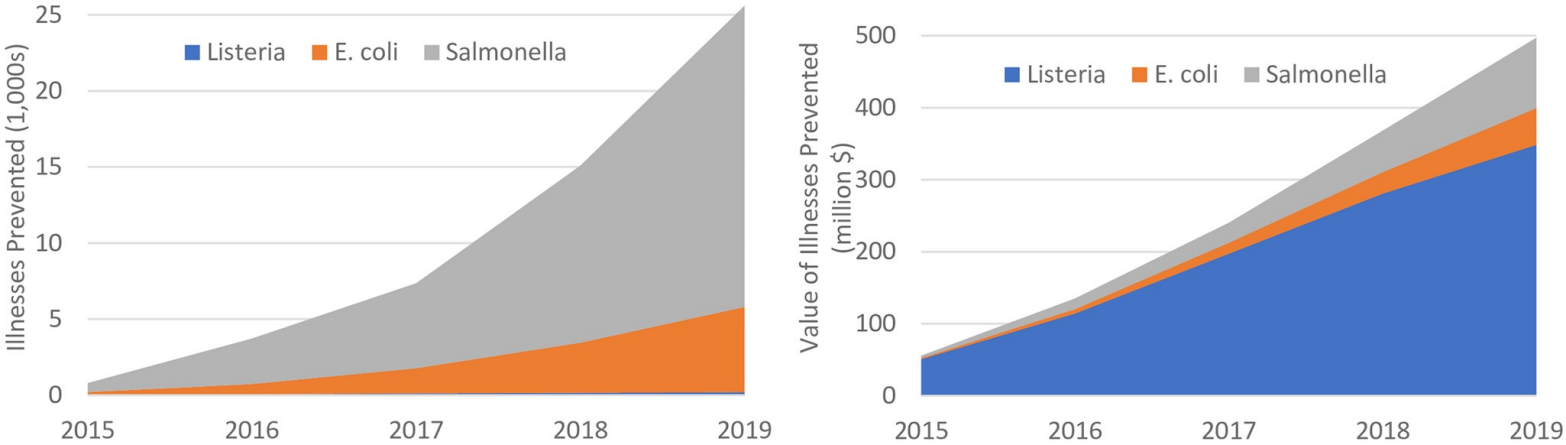
Estimated illnesses prevented per pathogen. Annual illnesses and associated monetary burden estimated to be prevented for *Listeria*, *E*. *coli*., and *Salmonella*; a graphical illustration of Table 4.

Fig 2 presents similar information graphically. The left side illustrates the annual mean estimated illnesses prevented by pathogen. Overwhelmingly, *Salmonella* accounts for the most illnesses prevented, about 77% of the total in 2019. This is due primarily to the higher number of unique *Salmonella* WGS isolates uploaded to NCBI PD by 2019 as compared to the other two pathogens (as illustrated in Fig 1) as well as the slightly higher under-reporting multiplier for observed *Salmonella* illnesses (presented in Table 3) [1]. In contrast, the right hand of Fig 2, which shows the associated monetary burden avoided, shows that averted *Listeria* illnesses account for the vast majority of estimated benefits, approximately 70% in 2019. This is especially interesting considering that *Listeria* illnesses accounted for only around 1% of the total estimated illnesses averted in that same year (shown in the left side of Fig 2). However, the result is not entirely unexpected, due to the higher mortality rate associated with *Listeria* illnesses, and in fact the program was targeted initially on *Listeria* illnesses for exactly this reason [6, 25–27].

**Table 3:** Benefits parameters.

Parameter	Description	Mean Estimates	90% CI	Data Source
WGS Reduction	Reduction in observable illnesses per 1,000 WGS NCBI isolates collected	6.09	(2.39, 9.79)	estimated
Under-reporting*	Multiplier to account for underreporting/underdiagnosis of illnesses			1
*Listeria*		2.31	(1.99, 2.62)	
*E*. *coli*		26.69	(16.25, 41.69)	
*Salmonella*		29.30	(22.63, 39.87)	
Monetary Loss	Value of loss due to single illness (in 2019 $)			25, 26, 27
*Listeria*		$1,661,269	($1,296,658, $2,138,712)	
*E*. *coli*		$9,125	($6,961, $11,782)	
*Salmonella*		$4,925	($4,078, $5,989)	

WGS, Whole Genome Sequence; NCBI, National Center for Biotechnology Information.

*For the estimates where we assume no underreporting/underdiagnosis, the multiplier is implicitly a value of 1.

The left side of Table 4 shows estimated illnesses and burden of illnesses averted if the multipliers for underreporting/underdiagnosis are omitted, essentially, making the conservative assumption that illnesses reported in the CORE database make up all illnesses associated with each of the outbreaks. Under these more restrictive assumptions the total burden of illness averted reaches nearly $50 million in year 2016 and over $150 million by 2019 by averting nearly 1,000 observed illnesses. The true magnitude of the effect of WGS source tracking on public health is likely somewhere in between these estimates, with and without the multiplier.

**Table 4:** Estimated burden of illness averted.

Observed Effects Only						With Underreporting and Underdiagnosis Multipliers				
	List.	E. coli	Sal.	Yearly Total	95% CI	List.	E. coli	Sal.	Yearly Total	95% CI
**Estimated Illnesses Averted**										
**2014**	2	0	3	5	(2–8)	4	13	80	98	(37–166)
**2015**	13	7	20	40	(16–64)	31	185	574	789	(297–1,339)
**2016**	30	25	102	157	(62–252)	69	671	2,982	3,722	(1,398–6,339)
**2017**	51	63	190	304	(119–489)	119	1,670	5,577	7,366	(2,770–12,534)
**2018**	73	123	397	593	(233–954)	169	3,281	11,636	15,085	(5,670–25,683)
**2019**	91	210	675	976	(383–1,569)	210	5,592	19,792	25,595	(9,619–43,589)
**Monetized Illnesses Averted in Millions of $**										
**2014**	$3.22	$0.00	$0.01	$3.24	($1.22–$5.51)	$7.43	$0.12	$0.39	$7.94	($2.96–$13.61)
**2015**	$22.07	$0.06	$0.10	$22.23	($8.36–$37.87)	$50.95	$1.68	$2.83	$55.46	($20.79–$94.89)
**2016**	$49.48	$0.23	$0.50	$50.21	($18.89–$85.49)	$114.23	$6.13	$14.69	$135.04	($51.03 -$229.39)
**2017**	$85.51	$0.57	$0.94	$87.01	($37.72–$148.09)	$197.39	$15.24	$27.46	$240.09	($90.87–$406.78)
**2018**	$121.56	$1.12	$1.96	$124.64	($46.92–$211.99)	$280.62	$29.94	$57.30	$367.86	($139.56–$620.41)
**2019**	$150.96	$1.91	$3.33	$156.19	($58.83–$265.47)	$348.48	$51.03	$97.47	$496.98	($188.62–$835.92)

List., *Listeria*; Sal., *Salmonella*; CI, Confidence Interval.

Underreporting/underdiagnosis multiplier as well as illness burden by pathogen reported in Table 3 [1, 25–27].

Current federal funding for the program, including funds supplied by federal and state health agency partners for lab set up costs, collection and testing costs, and internal costs to run the program is roughly $21.3 million per year. Results suggest the source tracking program likely broke even by year 2, and by 2019 the estimated net benefits are roughly $475 million. Even under the conservative scenario the estimated net benefits in 2019 are nearly $125 million.

## Discussion

Based on the theoretical implications of adopting WGS source tracking and surveillance, it is unclear, *a priori*, if the net observed effect would be driven by an uptick in identified illnesses or a decrease in total illnesses. Empirical results show a decrease in observed illnesses, while more outbreaks are identified with WGS source tracking, suggesting outbreaks are possibly being solved somewhat faster or smaller outbreaks are being detected more frequently.

A fully implemented program could cost anywhere between $10 and $50 million annually and could add upwards of 25 thousand new isolates each year, growing towards 7 million records in total. The expected health benefits of the program, measured in avoided illness, could grow into billions of dollars under a fully implemented WGS source tracking program in the US. Further, the marginal costs of collecting, sequencing and uploading isolates is likely to decrease significantly over time as the necessary technology becomes cheaper and once regional labs are established [28, 29].

Our results are in line with previous studies on similar programs. While Scharf et al. are estimating the effects of a fully implemented PulseNet program, the predecessor program to WGS, our results suggest that the WGS may attain a comparable level of benefits by 2020/21 [15]. Jain et al. estimate net benefits of the Canadian WGS program on *Salmonella* only, using two representative outbreaks, of between $5-$90 million [16]. The net benefits presented here, drawn from complete data on only *Salmonella* outbreaks investigated by FDA from 1999–2019, are more conservative and suggest that the benefits from *Salmonella* reduction would not approach $90 million dollars until 2019, five years after implementation of the program. Ford et al. and Alleweldt et al. recently published break-even analyses suggesting that WGS source tracking programs would need to prevent between 0.2% and 2% of illnesses linked to serotyped pathogens in Australia, Europe and the United States [30, 31]. Our results suggest this level of prevention is attainable even in the early stages of WGS source tracking implementation.

The estimated benefits in this study are largely driven by the reduction from *Listeria* illnesses. In 2014, averted *Listeria* illnesses accounted for 93.5% of the total estimated benefits; by 2019 this had fallen to 70%. The reasons for this are 1) the cost per illnesses for *Listeria* is much higher than the other WGS pathogens due to the high mortality rate associated with listeriosis, and 2) *Listeria* was the first pathogen to be targeted by the WGS source tracking program, because of the severe human health outcomes associated with it [6, 25, 32]. However, by 2019 the number of *Salmonella* and *E*. *coli* isolates collected had surpassed those for *Listeria*, leading to their increased role in the total benefits.

While NCBI PD data includes WGS records from FDA, CDC, USDA FSIS, industry, and academic labs, we note that the FDA-CORE dataset used for this analysis represents only a portion of foodborne outbreaks. This data represents outbreaks that rose to the level of an FDA investigation based on the suspected contaminated product, size of the outbreak, jurisdiction of the outbreak, and other factors. This data is not representative of all foodborne illnesses in the U.S., as it includes only a subset of outbreaks associated with FDA-regulated foods. This does not diminish the findings of this study regarding the effectiveness of WGS source tracking as a tool in outbreak identification and investigation.

Although our data does not yet have the predicative power to separate estimates by food source, or even individual pathogen, at this time, future research could examine the extent to which reductions for specific pathogen/food pair sub-populations are driving early estimated results. For example, approximately 70% of our total estimated benefits in 2019 are driven by a reduction in *Listeria* illnesses and more than 75% of *Listeria* illnesses have been linked to dairy products or fruits [11, 13, 33]. This may suggest that focusing efforts on these specific benefit drivers may yield improved outcomes in earlier stages of the program, a hypothesis that can be tested as additional data is generated. Studies such as this may also help to prioritize the sequencing and uploading of isolates from different pathogens beyond the three WGS source tracking program pilot pathogens (*E*. *coli*, *Listeria*, and *Salmonella*) studied here.

Similarly, estimates into the quality and genomic diversity within a particular set of NCBI PD isolates may yield information to further understand the impact we observe in this study on human health. For example, the number of links or closely related samples to a particular isolate or group of isolates could be disproportionately driving the benefits of the program. A disaggregated analysis of the database, employing network-effects, or a similar technique could prove beneficial and help better understand the most efficient deployment of resources to mitigate foodborne illness. FDA annually designs sampling plans for food pathogen commodity pairs that could direct priority surveillance for the agency and its domestic partners.

This analysis does not account for private investment in those food safety measures put in place due to information gained from the WGS source tracking program and that have been inherently captured in our benefits estimate. Further research should also examine the WGS source tracking program’s potential to save costs for industry as an outcome of smaller and more targeted recalls.

Finally, the methodology described in this paper may be used to analyze additional WGS surveillance data and other sources of preventable infectious diseases. As WGS source tracking technology is integrated into more labs and used to track more pathogens, further analyses will shed light on any additional benefits and can be used to help develop additional metrics to measure the progress of the continued WGS source tracking program.

## Conclusion

This study examines the effectiveness of the U.S.’ WGS source tracking program. Using data collected on outbreaks associated with FDA-regulated foods, we estimate the effect additional WGS NCBI PD isolates have on the burden of foodborne illness for *E*. *coli*, *Listeria*, and *Salmonella*. Results suggest that WGS source tracking has been successful. Illness numbers of heavily sequenced pathogens are falling faster relative to non-sequenced pathogens and observed outbreaks related to WGS source tracking program pathogens are getting smaller. Under current funding and growth levels, the net benefits are somewhere between $100 million and $450 million. These estimated benefits of the WGS source tracking program easily outweigh the estimated costs of implementation after the second year. Once the program is fully implemented, we may see net benefits measure in billions of dollars.

Other countries and different geographic regions have different baseline regulatory climates and food safety cultures, so the marginal effect of WGS source tracking may vary from what we observe in US data [30, 31], but this study provides strong evidence for a significant improvement in food safety anywhere WGS source tracking is implemented. WGS surveillance technology is transferable to other international food safety agencies and should be a part of capacity building and partnership programs. Applications of WGS source tracking are expected to expand rapidly and play critical roles in detection, surveillance, root cause analysis and potential prediction of future pandemics, outbreaks, and contamination events. We envision a global food shield and pathogen surveillance system with many countries sequencing and sharing the genomes of human, animal and plant pathogens [3, 6, 7, 11]. This integration of genomic data and metadata descriptions will advance the one health strategy for improving public health for all nations. The economic benefits detailed herein will help drive adoption of WGS surveillance globally. In addition, WGS source tracking has had a significant value in the battle against COVID-19 and likely will have future epidemic or pandemic scenarios [34]. The benefits of the program are applicable to COVID-19 and other infectious disease control applications (hospitals, nursing homes, medical manufacturing, waste management, composting, agricultural water use and reuse). In each of these instances, results from this analysis demonstrate that incorporating WGS source tracking may provide real positive public health benefits even in the early stages of implementation.

## Supporting information

S1 Appendix Supplementary material. Technical appendices.
(DOCX)

S1 Table Full regression results. (XLSX)

S1 Dataset Regression data. Full database used in this analysis.
(XLSX)

S1 File Readme. Readme file for regression data.
(TXT)
