# Supplementary material for: An economic evaluation of the Whole Genome Sequencing source tracking program in the U.S
Source: PLoS One. 2021 Oct 6;16(10):e0258262. doi: 10.1371/journal.pone.0258262 (PMC8494326; doi:10.1371/journal.pone.0258262)
Supplement: S1 Appendix — Technical appendices. (DOCX) [file pone.0258262.s001.docx]

# Supplementary Material

## S1. The Social Value Function

This section presents a stylized theoretical model that illustrates the potential effects of the WGS program and sets the stage for the empirical analysis. We model a partial social value function (SV) to describe the potential effects of the WGS program on the number of foodborne illness (observed and unobserved), public costs, as well as the effects of the program on firm behavior and production costs.

SV captures the net societal effects of the production of a valuable food commodity where there is a small but positive probability that food could be contaminated during production and cause a foodborne illness outbreak. We define SV below in Equation S1:

$SV\left( x,e \right)=\underset{profit function}{\underbrace{\left[ p_{x}*x-\left( c_{x}\left( x \right)+c_{e}\left( e \right) \right) \right]}}-\underset{\begin{aligned} public health externality \\ function \end{aligned}}{\underbrace{\left[ C_{I}*(x*\gamma_{I}\left( e \right)*n_{I}) \right]}}$ (S1)

The profit function of the representative firm captures the value of the goods produced to industry and consumers. The firm maximizes profits over production of $x$ to the constant per unit consumer price $p_{x}$ and the costs of production $c_{x}\left( x \right)$ that are a function of how much they produce and the unit costs of production. The firm also invests in the effectiveness of controls for potential food contamination, depicted in the model as *e*. As the firm invests more, the effectiveness of the controls increases. The cost of investment in controls $c_{e}\left( e \right)$ is increasing in *e*, but *e* does not affect revenue in this simple model. So profits decrease with respect *e*. Recall avoidance is implicit in the firm’s decision to invest in the baseline level of *e*, we can call this level $\bar{e}$. Consideration of current regulation is also implicit in $\bar{e}$. Profits decreasing in *e* implies that the firm would not invest above baseline levels of controls if they do not internalize any of the of the risk of illness. This isn’t true in general (some firms may advertise food safety measures to increase market share or charge a price premium), but for the purposes of illustrating the potential effects of WGS, it serves as a useful simplifying assumption.

The externality function captures effects not fully internalized by the profit maximizing firm, in this case, the potential effects of foodborne illness associated with food production. In the model, $\gamma_{I}\left( e \right)$ is the probability for any level of *e*, that a unit of production causes an outbreak. The probability, by design, decreases as the firm increases its investment in food safety controls. $n_{I}$ is the number of illnesses associated with any outbreak event, and $C_{I}$ is the marginal burden of illness.

Taking the partial derivatives of SV with respect to $\gamma_{I}$ and $e$ gives us the effect of these variables on the social value function:

$\frac{\partial SV}{\partial\gamma_{I}}= \underset{\begin{aligned} total burden of \\ an outbreak \end{aligned}}{\underbrace{-C_{I}*x*n_{I}}}<0$ (S2)

$\frac{\partial SV}{\partial e}= \underset{\Delta in cost}{\underbrace{\left[ -\frac{\partial c_{e}}{\partial e} \right]}}-\underset{\Delta in burden of illness}{\underbrace{\left[ C_{I}*x*\frac{\partial\gamma_{I}}{\partial e}*n_{I} \right]}}⋛0$ (S3)

Equation S2 demonstrates the intuitive result that SV is decreasing in $\gamma_{I}$, so society is better off as the marginal probability of an outbreak occurring decreases. However, Equation S3 demonstrates that the sign of the effect of $e$ on SV is indeterminate. The result informs us in two ways. First, additional investment in $e$ is only socially optimal if the cost of additional investment is outweighed by the additional public health benefits accrued by more investment in control effectiveness. Second, if a firm does not fully internalize the potential effect of their investment in $e$ on public health, they will likely underinvest in control effectiveness.

There are reasons a firm may not fully internalize the risk of contamination leading to public illness. First, many illnesses are never specifically identified and traced back to the origin of contamination. So, a firm that produces contaminated food may not know they have a food safety issue, or the extent of the problem. Second, in the rare case of bad actors, the probability of production causing illness *and* any single issue being traced back to a particular firm is small, so a firm may not fully invest in expensive controls if they believe they will not be implicated.

Next, we add WGS implementation to SV. WGS enters the SV function in four places. First, WGS will give the firms information of about their process and sheds light on problems they may have. In this way, WGS will provide an incentive for system change and increase investment in control effectiveness. Second, the change in investment, due to WGS implementation, will affect the probability of an outbreak or illness event. Third, WGS is expected to directly decrease the cost of outbreaks or illness occurrences by allowing for faster, more efficient tracing of the cause of illness, decreasing the number of illnesses in any given outbreak. Last WGS will have direct implementations costs. The full model is shown below in equation S4:

$SV\left( x,e, WGS \right)=\underset{profit function}{\underbrace{\left[ p_{x}*x-\left( c_{x}\left( x \right)+c_{e}\left( e(WGS \right) \right) \right]}}-\underset{public health externatility function}{\underbrace{\left[ C_{I}*x*\gamma_{I}\left( e\left( WGS \right) \right)*n_{I}\left( WGS \right) \right]}}-\underset{implentation cost}{\underbrace{\left[ c_{WGS}\left( WGS \right) \right]}}$ (S4)

The SV function is now a function of the control variable, *WGS*. We define the firm’s level of investment in the effectiveness of food safety controls, $e(WGS)$, as an increasing function of $WGS$. This term also affects the probability, $\gamma_{I}\left( e\left( WGS \right) \right)$, of an outbreak occurring. The number of illnesses associated with an outbreak, $n_{I}(WGS)$, is a decreasing function of $WGS$. And a new term is added,$c_{WGS}(WGS)$, that captures the direct implementation costs of WGS.

To see the effect of $WGS$ on social welfare, take the partial derivative of *SV* with respect to $WGS$.

$\frac{\partial SV}{\partial WGS}= -\underset{\Delta in cost}{\underbrace{\left[ \frac{{\partial c}_{e}}{\partial e}*\frac{\partial e}{\partial\omega WGS} \right]}}-\underset{\Delta in burden of illness}{\underbrace{\left[ C_{I}*x*\left( \left( \frac{\partial\gamma_{I}}{\partial e}*\frac{\partial e}{\partial\omega WGS} \right)*n_{I}(\omega WGS)+\gamma_{I}\left( e\left( \omega WGS \right) \right)*\frac{{\partial n}_{I}}{\partial WGS\omega} \right) \right]}}-\underset{\begin{aligned} \Delta in \\ program \\ cost \end{aligned}}{\underbrace{\left[ \frac{\partial c_{WGS\omega}}{\partial WGS\omega} \right]}}⋛0$ (S5)

The first set of terms are the effect of WGS on the firm’s cost function. As WGS is implemented, and the library of isolates expands, root cause analysis becomes viable in more cases, and firms may be compelled to take on process changes and invest more food safety controls, increasing costs. Increased societal costs are also directly incurred through implementation of the program, shown in the last set of terms. The effect of these costs on *SV* is negative. The effect of WGS on the total societal burden of foodborne illness associated with food production is shown in the second set of terms. WGS will decrease the number illnesses, both directly by increasing the efficiency of traceback efforts, and indirectly by increasing private investment in control effectiveness. The total effect of WGS on social welfare is ambiguous. We cannot know without at least some cost benefit analysis, weighing the estimated benefits of illness reduction against the estimated costs of implementation, whether society as a whole is better off with WGS.

## S2. Observed and Unobserved Illnesses

Now consider the reality that many illnesses go undetected. Equation S6 shows the case where there is some probability associated with whether an illness is detected or observed.

$n_{I}\left( WGS \right)=\underset{\begin{aligned} number of illnesses \\ per outbreak \end{aligned}}{\underbrace{n_{I}\left( WGS \right)}}*\underset{\begin{aligned} cumulative probability an outbreak \\ is observed/unobserved \end{aligned}}{\underbrace{\left( \alpha_{O}(WGS)+\alpha_{U}(WGS) \right)}}$ , where: $\alpha_{O}+\alpha_{U}=1$ (S6)

Now all foodborne illnesses associated with food production are split into “observed/det” and “unobserved/undetected” by $\alpha_{O}(WGS)$, the probability that an illness is observed and $\alpha_{U}(WGS)$, the probability that an illness is unobserved/undetected.

One additional effect of implementation is that WGS will identify outbreaks in smaller clusters and tie more illnesses to identified sources. So even if we hold the effect of WGS on total illness constant, WGS will increase the probability that an illness is detected/observed and decrease the probability that an illness is undetected.

Plugging in Equation S6, the illness function for all foodborne illnesses becomes:

$I_{A}=\underset{\begin{aligned} probability outbreak \\ occurs \end{aligned}}{\underbrace{x*\gamma_{I}\left( e\left( WGS \right) \right)}}*\underset{\begin{aligned} number of illnesses \\ per outbreak \end{aligned}}{\underbrace{n_{I}\left( WGS \right)}}*\underset{\begin{aligned} cumulative probability an outbreak \\ is observed/unobserved \end{aligned}}{\underbrace{\left( \alpha_{O}(WGS)+\alpha_{U}(WGS) \right)}}$ (S7)

In Equation S5, we showed that WGS, by design, decreases the total number of foodborne illnesses. However, in empirical analysis, we can only study the effect of the WGS program on what we observe, so it is useful to isolate the effect of WGS on observed illnesses.

The number of observed illnesses can be written as the product of the total number of illnesses and the probability that an illness is observed: $n_{I}\left( WGS \right)*\alpha_{O}(WGS)$. The illness/externality function in terms of observed illnesses becomes:

$I_{O}=\underset{\begin{aligned} probability outbreak \\ occurs \end{aligned}}{\underbrace{x*\gamma_{I}\left( e\left( WGS \right) \right)}}*\underset{\begin{aligned} number of illnesses \\ per outbreak \end{aligned}}{\underbrace{n_{I}\left( WGS \right)}}*\underset{\begin{aligned} probability illnesses \\ are observed \end{aligned}}{\underbrace{\alpha_{O}(WGS)}}$ (S8)

The effect of WGS on the number of observed illnesses is shown in Equation S9:

$\frac{\partial I_{O}}{\partial WGS}= x*\left( \underset{\begin{aligned} \Delta in probability of \\ an outbreak \end{aligned}}{\underbrace{\left[ \left( \frac{\partial\gamma_{I}}{\partial e}*\frac{\partial e}{\partial\omega WGS} \right)*n_{I}*\alpha_{O} \right]}}+\underset{\begin{aligned} \Delta in number of illnesses \\ per outbreak \end{aligned}}{\underbrace{\left[ \gamma_{I}*\frac{\partial n_{I}}{\partial WGS\omega}*\alpha_{O} \right]}}+\underset{\begin{aligned} \Delta in probability of outbreak \\ identification/detection \end{aligned}}{\underbrace{\left[ \gamma_{I}*n_{I}*\frac{\partial\alpha_{O}}{\partial WGS\omega} \right]}} \right)⋛0$ (S9)

The sign of the effect of *WGS* on the number of observed illnesses is indeterminant. The effect of *WGS* on investment in controls and subsequent effect on the probability that an outbreak occurs, and the direct of effect of *WGS* on the average number of outbreak illnesses both have a negative effect on the number of observed illnesses. However, the effect on the probability that an illness is detected has a positive effect on the number of observed illnesses. If the effect of *WGS* on the probability of all illnesses occurring dominates, the sign is negative, and we should observe a decline in illnesses. However, if the effect of WGS on detection dominates, then we would observe an increase in illnesses even as the total falls. Especially in the short run, because the effects of the program on investment in controls to be longer term, the effect of detecting more illnesses could drive the results of any regression of number of illnesses on WGS implementation. Even if we find that WGS reduces the number of illnesses, the estimate will be dampened by this effect.

## S3. Stata Code

import excel using "PLOSfulldata.xlsx", firstrow

xtset Year pathid

********************

**** regression ****

********************

local pathfe adverse bacill bruce cigua clost crono crypto cyclo fungus hepa myco noro para shig staph tetro vibrio

local FSMA ppcrule fpcrule ipcrule

local commfe dairy dietsup egg fish infant multiple produce shellfish unknown

local control `pathfe' `commfe' y1-y4 y6-y14 y16-y21

local controlfsma `pathfe' `commfe' y1-y4 y6-y14 y19-y21

local dnd inter wgsdum listeria campy ecoli salmonella

**** illnesses ****

reg illnesses wgslib, r cluster(pathid)

reg illnesses wgslib `control', r cluster(pathid)

reg illnesses wgslib `controlfsma' `FSMA', r cluster(pathid)

**** outbreak ****

reg outbreak wgslib, r cluster(pathid)

reg outbreak wgslib `control', r cluster(pathid)

reg outbreak wgslib `controlfsma' `FSMA', r cluster(pathid)

**** average ill ****

reg avgill wgslib, r cluster(pathid)

reg avgill wgslib `control', r cluster(pathid)

reg avgill wgslib `controlfsma' `FSMA', r cluster(pathid)

clear

## S4. The Benefits Simulation in @Risk (Version 8)

To estimate Equation 6:

$Benefits=\hat{\beta}_{1} x WGS Isolates x Underreporting Multiplier x Monetary Loss$ (6)

We start with the estimated reduction in observable illnesses per 1,000 WGS isolates collected, $\hat{\beta}_{1}$ in Equation 6:

| Table S4-1. WGS Reduction | |  |  |  |
| --- | --- | --- | --- | --- |
| **Parameter** | **Description** | **Distribution** | | |
| WGS Reduction | Reduction in observable illnesses per 1,000 WGS isolates collected | "@RiskNormal(-6.09, 2.25)" | | |

Next we multiply $\hat{\beta}_{1}$ by the number of WGS isolates in the library by pathogen, by year:

| Table S4-2. WGS Isolates Collected (in Thousands) | | | |
| --- | --- | --- | --- |
|  | ***Listeria*** | ***E. coli*** | ***Salmonella*** |
| 2014 | 0.32 | 0.08 | 0.45 |
| 2015 | 2.18 | 1.14 | 3.21 |
| 2016 | 4.89 | 4.13 | 16.71 |
| 2017 | 8.45 | 10.27 | 31.25 |
| 2018 | 12.01 | 20.18 | 65.19 |
| 2019 | 14.92 | 34.40 | 110.89 |

The result is the predicted observed reduction in illnesses due to accumulation of WGS isolates by pathogen, by year. This is multiplied by the underreporting/underdiagnosis multiplier shown in Table S4-3 to estimate full reduction in illnesses.

| Table S4-3. Underreporting/Underdiagnosis Multiplier | | |
| --- | --- | --- |
| **Parameter** | **Description** | **Distribution** |
| Under-reporting | Multiplier to account for underreporting/underdiagnosis of illnesses |  |
| *Listeria* |  | @RiskBetaGeneral(3, 2.9, 1.8, 2.8) |
| *E. coli* |  | @RiskBetaGeneral(1.2, 2.7, 15, 53) |
| *Salmonella* |  | @RiskBetaGeneral(1.3, 4.8, 21.8, 57) |
| To make the more conservative estimate in the paper, we set the multiplier equal to one | | |

The result is the total estimated reduction in illnesses due to accumulation of WGS isolates. This is multiplied by the average (equal weight) of burden of illness estimates from the literature, by pathogen, using distributions shown in Table S4-4:

| Table S4-4. Monetary Burden of Illness Estimates | | | |
| --- | --- | --- | --- |
| **Parameter** | **Description** | **Distribution** | **Data Source** |
| Monetary Loss | Value of loss due to single illness (in 2014 $) |  | 25 |
| *Listeria* |  | @RiskBetaGeneral(2.3,2.5,1445112,1468384) |  |
| *E. coli* |  | ‘@RiskBetaGeneral(2.1,2.0,7814,12668) |  |
| *Salmonella* |  | ‘@RiskBetaGeneral(1.8,2.0,4868,5915) |  |
| Monetary Loss | Value of loss due to single illness (in 2012 $) |  | 26 |
| *Listeria* |  | ‘@RiskBetaGeneral(1.8,4.0,81000,3904000) |  |
| *E. coli* |  | ‘@RiskBetaGeneral(1.8,4.0,3447,23513) |  |
| *Salmonella* |  | ‘@RiskBetaGeneral(1.9,4.0,1558,10042) |  |
| Monetary Loss | Value of loss due to single illness (in 2014 $) |  | 27 |
| *Listeria* |  | 1,619,731 |  |
| *E. coli* |  | 4,035 |  |
| *Salmonella* |  | 3,221 |  |
| Distributions around burden of illness estimates could not be recreated for one of the papers [27], so point estimates were used. | | | |

The result is the weighted avoided monetary loss due to the accumulation of WGS isolates. We multiply by the GDP deflator to put the estimates in terms of 2019 constant dollars.

The simulation is run in @Risk (version 8) over 100,000 iterations.
